# Stimulated Brillouin Scattering in an AlGaN Photonics Platform Operating in the Visible Spectral Range

**DOI:** 10.1038/s41598-018-33183-7

**Published:** 2018-10-04

**Authors:** Francesco De Leonardis, Richard A. Soref, Mohammad Soltani, Vittorio M. N. Passaro

**Affiliations:** 1 Dipartimento di Ingegneria Elettrica e dell’Informazione, Politecnico di Bari, Via Edoardo Orabona n. 4, 70125 Bari, Italy; 2 Department of Engineering, The University of Massachusetts, Boston, Massachusetts 02125 USA; 3 Raytheon BBN Technologies, 10 Moulton Street, Cambridge, MA 02138 USA

## Abstract

We present Stimulated Brillouin Scattering (SBS) process in AlGaN integrated photonic waveguides. The wide bandgap of this III-Nitride material platform allows operating at visible wavelengths enabling large Stokes shifts. For this study, we employ a multiphysics approach that includes electric-photoelastic, magnetic-photoelastic, material interface displacement effects, and for optimal waveguide dimensions to find the Brillouin-active acoustic modes involved in the SBS process. The SBS power gain and the Stokes frequency shift are investigated for both backward and forward scattering processes, and it is shown that stokes shift larger than 50 GHz with high gain are achievable. Moreover, a parametric analysis is presented in order to demonstrate the possibility of realizing Brillouin lasers operating at blue wavelengths.

## Introduction

Stimulated Brillouin Scattering (SBS) is a third-order nonlinear process that produces efficient coupling between a traveling pump wave and induced acoustic modes^1,2^. This nonlinear effect has been widely studied for several applications such as frequency conversion^3–5^, radio frequency signal processing^6–8^, slow light^9–13^, distributed temperature sensing^14^, novel lasers sources^15–19^, and gyroscope sensors^20^. A recent article reviews the promises and challenges of SBS on chip-scale photonic integrated circuits^21^. Currently, many such on-chip experiments are based on soft-glass waveguides with substantial SBS gains of the order of 13 dB/cm per Watt of pump power at 1.55 µm. These glasses remain very suitable for experiments at larger wavelengths. However, their total device gain is reduced because of the greater effective mode area at longer wavelengths, thus resulting in lower Stokes intensities for a given pump power. It is generally recognized that waveguide structures with higher confinement and/or nano-scale sizes provide stronger photon-phonon coupling, and thereby larger power gain. For this reason, the SBS effect in group IV semiconductors (Si, Ge and, more generally, the crystal alloy SiGeSn) are receiving increased attention for practical applications, both in the near-infrared (NIR, i.e. 1.31–1.55 µm) and mid-infrared (MIR, i.e. 1.8 to 5.0 µm), with a caveat that the Stokes shift is small due to the operation at longer wavelength.

The versatility of Brillouin processes depends on the ability to understand and engineer the photon-phonon coupling. Over the past several decades, various conceptually simple and useful methods have been employed to predict the strength of SBS coupling within guided-wave systems based on modal overlap integrals. Although these treatments have proven remarkably accurate for the prediction of SBS in optical fibers, they cannot be applied in nano-scale size waveguides where the vectorial nature of the optical and acoustic modes involved in the scattering process induce significant electrostriction and boundary radiation- pressures effects. To better take into account these effects, a general method of calculating SBS gain and Stokes shift has been recently proposed^22,23^.

Armed with these advances in accurate SBS modelling for submicron waveguides, much attention has been recently devoted to suspended silicon waveguides with very small cross sections as a technological solution to induce extraordinarily high SBS gains, due to a combination of small modal area and high radiation pressure effects^22^. Although this approach could clearly provide extremely efficient SBS-devices, these waveguides typically have to be completely or nearly completely suspended over long distances to ensure optical and acoustic mode confinement, resulting in mechanically fragile devices and difficult fabrication. For these reasons, Ge waveguides buried in silicon nitride have been recently proposed^24^ as an efficient candidate to realise high SBS power gain in the MIR spectrum range.

III-Nitride materials including Al_x_Ga_1−x_N can be another promising integrated photonic platform that allow operation at shorter wavelengths due to their wide bandgap properties, thereby, enabling SBS with much larger Stokes shift. One outcome of such large Stokes shift can be the generation of low noise high frequency microwave and millimetre wave signals, when beating the pump laser with the SBS signal at the photodetector.

The purpose of this paper is to study SBS process in an Al_x_Ga_1−x_N waveguide in the visible range for the aforementioned promises. To the best of our knowledge, exploring the SBS effect in the visible spectral range has not yet been developed in the literature. Thus, we address here the relevant physics in order to engineer on-chip Brillouin power gain and Stokes shift based on the Al_x_Ga_1−x_N technological platform.

The paper is organized as follows. The fundamental equations governing the SBS gain and Stokes shift are presented in the Theory Section. Then in the Result Section we apply the presented theory to Al_x_Ga_1−x_N waveguide to calculate their SBS effect for an operation in the visible spectrum. Detailed parametric simulations that include changing the waveguide sizes and material distribution will be investigated. As an application study, the feasibility of Brillouin laser in the blue wavelength on this waveguiding platform is investigated. Finally, the Conclusion Section summarizes our findings with concluding remarks.

## Theory

We present a parametric investigation to determine specific waveguided architectures able to realise a Brillouin power gain comparable with the values obtained with the standard technological platforms operating in the NIR and MIR spectrum regions, such as silicon, germanium, and As_2_S_3_.

**Figure 1:**
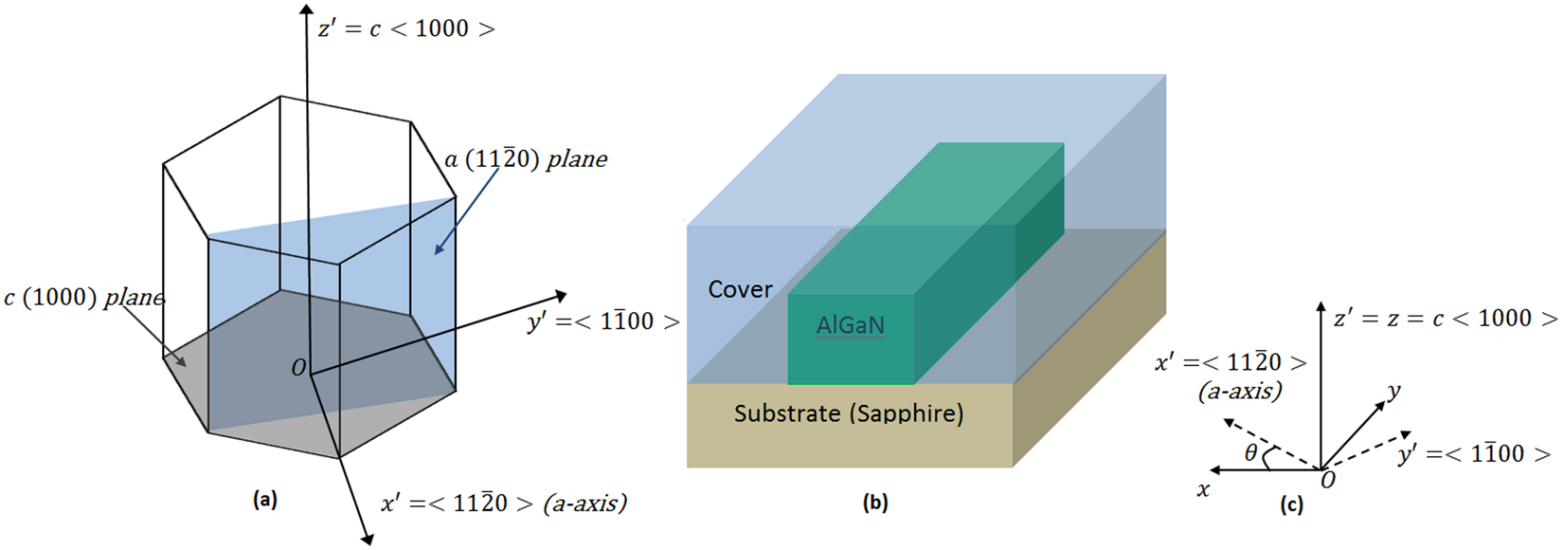
(**a**) Wurtzite structure in the crystallographic coordinate system (*Ox*′*y*′*z*′); (**b**) Cross section of an AlGaN Waveguide on a Sapphire substrate; AlGaN and sapphire are wurtizite crystals; (**c**) Waveguide frame of reference (Oxyz) and crystallographic axes.

The characteristic planes and axes of the wurtzite crystallographic structure (AlN, GaN, and Al_x_Ga_1−x_N) are shown in Fig. 1(a). We note that most of nitride devices are available only as bulk crystalline wafers cut in on-axis or off-axis orientations. The on-axis-orientation cut results in a wafer with its c-axis perpendicular to its surface, with the ordinary and extraordinary refractive indices in the plane of the wafer, and perpendicular to the plane of the wafer, respectively. As evidenced clearly in^25^, this cut is ideal for photonic devices, since the TE polarization is aligned with the crystal ordinary axis and the TM polarization is aligned with its extraordinary axis, thereby preventing unwanted polarization rotation. Thus, we assume in general that the waveguide global system (*Oxyz*) is rotated by an *θ* angle with respect to the crystallographic system (*Ox*′*y*′*z*′, local system). Then, the SBS power gain is related to the overlap between the confined acoustic and optical modes involved in the Brillouin scattering process. According to the theory proposed in^23^, we can express the optical field as a superposition of two eigenmodes (pump, and Stokes):

1 $${\boldsymbol{E}}={\tilde{{\boldsymbol{e}}}}_{{\boldsymbol{p}}}(x,z){a}_{p}(y,t){e}^{j({\beta }_{p}y-{\omega }_{p}t)}+{\tilde{{\boldsymbol{e}}}}_{{\boldsymbol{S}}}(x,z){a}_{S}(y,t){e}^{j({\beta }_{S}y-{\omega }_{S}t)}+c.\,c$$

where *c.c*. and *a*_*i*_(*y*, *t*) (with *i* = *p*, *S*) indicate the complex conjugate terms, and the slowly varying envelope functions, respectively. The functions ${\tilde{{\boldsymbol{e}}}}_{{\boldsymbol{i}}}(x,z)$ represent the spatial mode distributions, solutions of the Helmholtz equation with wave vector $\hat{{\boldsymbol{y}}}{\beta }_{i}$, angular frequency *ω*_*i*_, in the global frame of reference (*Oxyz*). Similarly, the acoustic modes can be written as:

2 $${\boldsymbol{U}}=\tilde{{\boldsymbol{u}}}(x,z)b(y,t){e}^{j(qy-{\Omega }_{B}t)}+c.\,c$$

where *q*, *Ω*_*B*_, and *b*(*y*, *t*) represent the acoustic wave vector, the angular eigenfrequency and the slowly varying envelope function, respectively. The function $\tilde{{\boldsymbol{u}}}(x,z)$ is the spatial distribution of the mechanical displacement vector and it is solution of the following eigenvalue problem:

3 $$\rho {{\rm{\Omega }}}_{B}^{2}{\mathop{u}\limits^{ \sim }}_{i}+\sum _{ijkl}{({{\rm{\nabla }}}_{T}+jq\hat{{\boldsymbol{y}}})}_{j}{c}_{ijkl}{({{\rm{\nabla }}}_{T}+jq\hat{{\boldsymbol{y}}})}_{k}{\mathop{u}\limits^{ \sim }}_{l}=0$$

here *i*, *j*, *k*, *l* = (*x*, *y*, *z*), *ρ*, and $\bar{{\boldsymbol{c}}}$ are the material density and the stiffness tensor, respectively.

Finally, the Brillouin power gain can be calculated by means of:

4 $${g}_{B}=\,\frac{2{\omega }_{p}{{\rm{\Omega }}}_{B}{|Q|}^{2}}{\alpha {P}_{p}{P}_{S}{P}_{b}}$$

where *P*_*p,S*_ and *P*_*b*_ are the pump (Stokes) and acoustic modal power^23^, respectively. Moreover, the term *α* denotes the acoustic decay parameter, depending on the dynamic viscosity tensor $\bar{{\boldsymbol{\eta }}}$^23^. The acoustic-optic overlap *Q* can be calculated as  = *Q*^(*ePE*)^ + *Q*^(*mPE*)^ + *Q*^(*MB*)^, where the first, second and third term represent the contributions induced by the electric-photoelastic, magnetic-photoelastic, and material interface displacement effects, respectively^23^:

5 $${Q}^{(ePE)}={\varepsilon }_{0}{\varepsilon }_{r}^{2}\int d{r}^{2}\sum _{ijkl}{[{\mathop{e}\limits^{ \sim }}_{S,i}]}^{\ast }{\mathop{e}\limits^{ \sim }}_{p,j}{p}_{ijkl}{{\rm{\partial }}}_{k}{\mathop{u}\limits^{ \sim }}_{l}^{\ast }$$

6 $${Q}^{(mPE)}=j{\rm{\Omega }}{\mu }_{0}{\varepsilon }_{0}({\varepsilon }_{r}-1)\int d{r}^{2}({\mathop{{\boldsymbol{e}}}\limits^{ \sim }}_{p}\times {[{\mathop{{\boldsymbol{h}}}\limits^{ \sim }}_{S}]}^{\ast })\cdot {\mathop{{\boldsymbol{u}}}\limits^{ \sim }}^{\ast }$$

7 $${Q}^{(MB)}={\int }_{C}dr(\hat{{\boldsymbol{n}}}\cdot {\tilde{{\boldsymbol{u}}}}^{\ast })[{\varepsilon }_{0}({\varepsilon }_{a}-{\varepsilon }_{b}){({\tilde{{\boldsymbol{e}}}}_{S}\times \hat{{\boldsymbol{n}}})}^{\ast }\cdot ({\tilde{{\boldsymbol{e}}}}_{p}\times \hat{{\boldsymbol{n}}})-\,{\varepsilon }_{0}^{-1}({\varepsilon }_{b}^{-1}-{\varepsilon }_{a}^{-1}){({\tilde{{\boldsymbol{d}}}}_{S}\cdot \hat{{\boldsymbol{n}}})}^{\ast }({\tilde{{\boldsymbol{d}}}}_{p}\cdot \hat{{\boldsymbol{n}}})]$$

where ${\tilde{{\boldsymbol{d}}}}_{i}$, and ${\tilde{{\boldsymbol{h}}}}_{i}$ (with *i* = *p*, *S*) are the electric induction field and the magnetic field distributions, respectively. In Eqs (5) and (6) the integrals are carried out over the whole transversal plane of the waveguide. On the contrary, the integral in Eq. (7) is a line integral to be carried out along all boundaries with normal vector $\hat{{\boldsymbol{n}}}$ between different materials with relative permittivities *ε*_*a*_, and *ε*_*b*_, respectively.

## Results

In this section and using the theoretical approach presented above, we evaluate both the Brillouin power gain and the Stokes shift in optical waveguides in wurtzite-Al_x_Ga_1−x_N waveguides. In our analysis the waveguide sits either on a sapphire substrate or suspended. We consider two pump wavelengths of 450 nm and 780 nm for our analysis due to the maturity of coherent semiconductor lasers at these wavelengths, and will specify for each waveguide structure the wavelength that is used. Using the Voigt notation in the crystallographic frame of reference system (*Ox*′*y*′*z*′), the wurtzite structure admits only five independent stiffness coefficients and twelve non-zero *c*′_*ij*_ elements. The same form holds for the photoelastic (*p*′_*ij*_) and dynamic viscosity (*η*′_*ij*_) tensors, respectively. Moreover, it is worth outlining that sapphire material, used as substrate in our investigations, generally crystallizes in a trigonal system, having six independent stiffness coefficients and eighteen non-zero zero *c*′_*ij*_ elements^26^. The physical parameters used in our simulations are listed in Table 1.

**Table 1:** Values of material parameters.

Parameters	Unit	AlN (wurtzite)	GaN (wurtzite)	Sapphire	Alumina (*)
Density, ρ	kg/m^3^	3255	6150	3980	~3980
**Stiffness tensor element** ***c*** **′**_***ij***_					
*c*′_11_	GPa	410	390	497.35	*E*(1 − *ν*)/[(1 + *ν*)(1 − 2*ν*)]
*c*′_12_	GPa	149	145	163.97	*Eν*/[(1 + *ν*)(1 − 2*ν*)]
*c*′_13_	GPa	99	106	112.20	*Eν*/[(1 + *ν*)(1 − 2*ν*)]
*c*′_14_ = *c*′_56_ = −*c*′_24_	GPa	0	0	−23.58	0
*c*′_33_	GPa	389	398	499.11	*E*(1 − *ν*)/[(1 + *ν*)(1 − 2*ν*)]
*c*′_44_ = *c*′_55_	GPa	125	105	147.39	*E*/(1 + *ν*)
*c*′_66_ = 0.5(*c*′_11_ − *c*′_12_)	GPa	125	122.5	166.69	*E*/(1 + *ν*)
**Photoelastic tensor element** ***p*** **′**_***ij***_					
*p*′_11_	—	−10 × 10^−2^	−8.6 × 10^−2^	−0.23	−0.23
*p*′_12_	—	−2.7 × 10^−2^	−2.3 × 10^−2^	−0.03	−0.03
*p*′_13_	—	−1.9 × 10^−2^	−1.7 × 10^−2^	0.02	−0.03
*p*′_14_ = *p*′_56_ = −*p*′_24_	—	0	0	0	0
*p*′_33_	—	−10.7 × 10^−2^	−9.1 × 10^−2^	−0.2	−0.23
*p*′_44_ = *p*′_55_	—	−3.2 × 10^−2^	−2.7 × 10^−2^	−0.1	−0.1
*p*′_66_ = 0.5(*p*′_11_ − *p*′_12_)	—	−3.7 × 10^−2^	−3.2 × 10^−2^	−0.1	−0.1

(*) The stiffness tensor elements for amorphous alumina can be evaluated as a function of the Young’s modulus, *E* = 345 GPa and Poisson ratio ν = 0.29.

Moreover, according to^27^, the generic physical parameter *R* of the Al_x_Ga_1−x_N alloy can be estimated by means of a linear interpolation as:

8 $$R(A{l}_{x}G{a}_{1-x}N)=x\cdot R(AlN)+(1-x)\cdot R(GaN)$$

In case of bulk material and under the plane wave approximation, the Stokes shift and the Brillouin scattering depend on the elastic tensor elements. In particular, the frequency shift is given by ${\Omega }_{B}=\pm \,2{\omega }_{p}n{v}_{s}sin(\vartheta /2)/{c}_{0}$, where *ϑ* is the angle between the incident pump wave and the scattered radiation from the acoustic wave and *n* is the bulk Al_x_Ga_1−x_N refractive index evaluated at the pump angular frequency *ω*_*p*_. The speed *v*_*s*_ of the longitudinal (transverse) acoustic phonon can be determined as ${v}_{s}=\sqrt{c{^{\prime} }_{11(44)}/\rho }$. The values listed in Table 1 and Eq. (8) give a Stokes shift ranging from 80 to 100 GHz, depending on the pump wavelength, for backward Brillouin scattering at visible wavelengths. However, we expect significant deviations in shift frequency in the case of nanoscale waveguides due to the vectorial nature of optical and acoustic modes. In the waveguide structures, we assume that the local coordinate system (*Ox*′*y*′*z*′) is correlated with the substrate crystalline axes, whereas the waveguide global system (*Oxyz*) is rotated by an *θ* angle with respect to (*Ox*′*y*′*z*′). In this sense, the fourth-rank stiffness tensors *c*′_*tuvw*_ (listed in Table 1) is transformed into *c*_*ijkl*_ (frame of reference (*Oxyz*)) on the basis of the direction matrix *a*_*mn*_:

9 $${c}_{ijkl}={a}_{it}{a}_{ju}{a}_{kv}{a}_{lw}c{^{\prime} }_{tuvw}$$

where:

10 $${a}_{mn}=[\begin{array}{ccc}cos(\theta ) & -sin(\theta ) & 0\\ sin(\theta ) & cos(\theta ) & 0\\ 0 & 0 & 1\end{array}]$$

### Backward SBS

Depending on the launching conditions, SBS can be categorized as forward SBS (FSBS) or backward SBS (BSBS). In BSBS, pump and Stokes waves propagate along opposite directions, generating axially-varying optical forces that excite traveling-wave acoustic modes. Considering both pump and Stokes waves with the same polarization and spatial distribution, we can approximate the Stokes mode as the time-reversed pump mode: ${\tilde{{\boldsymbol{e}}}}_{S}\approx {[{\tilde{{\boldsymbol{e}}}}_{p}]}^{\ast }$; *β*_*S*_ ≈ −*β*_*p*_; *q* ≈ 2*β*_*p*_; *P*_*S*_ ≈ −*P*_*p*_. Moreover, as outlined in^24^, the ideal material for the BSBS-active waveguide must be soft, dense and with high refractive index. Good acoustic confinement and consequently high power gain can be achieved if the sound velocity of the BSBS- active acoustic mode is smaller than the phase velocity of any other acoustic wave coupled into the structure:

11 $$\sqrt{{c}_{11}(core)/\rho } < max\{\sqrt{{c}_{11}(substrate)/\rho },\sqrt{{c}_{11}(cover)/\rho }\}$$

Our preliminary investigations is in Table 2 wherein a qualitative SBS performances of different waveguide structures has been summarized. The comparison in Table 2 shows that the Al_x_Ga_1−x_N on sapphire and the suspended Al_x_Ga_1−x_N waveguide platforms are good candidates to induce Brillouin effect at shorter wavelengths in the visible range (450 nm in our study), while GaN on Sapphire is more suitable for 780 nm.

**Table 2:** Qualitative performances for different waveguiding strcuctures.

Waveguide structure	Brillouin Active	Comments
Sapphire/AlN	No (Eq. 11)	No SBS process
Sapphire/Al_x_Ga_1−x_ N	Yes	High BSBS gain Stokes shift ~50 GHz; negligible TPA for x≥0.65, Defective AlGaN (due to lattice mismatch)
AlN/Al_x_Ga_1−x_ N	Yes	Weakly defective AlGaN, Very low BSBS gain
Sapphire /AlN/Al_x_Ga_1−x_N	Yes	Weakly defective AlGaN for AlN thickness ~1 µm; Very low BSBS gain
Sapphire/GaN	Yes	High BSBS gain, TPA effect at 450 nm No TPA effect at 780 nm
Suspended Al_x_Ga_1−x_N (with air pocket on SiO_2_)	Yes	High BSBS gain and moderate FSBS gain; Not suitable for long interaction length

Figure 1(b) shows a fully-etched waveguide cross-section with a width, *W*, and an height, *H*, used in the investigation of the SBS effect based on the Al_x_Ga_1−x_N strip waveguides. The substrate has been considered as crystalline Sapphire, while the cover material has been assumed as amorphous alumina or air. The Al_x_Ga_1−x_N platform sketched in Fig. 1(b) suffers from some limitations on the available alloy composition (x), as given by Eq. (11). Using the values listed in Table 1 and Eqs (8–11), we find that the strip Al_x_Ga_1−x_N waveguide can be BSBS-active for alloy concentration satisfying the condition x < 0.84. Thus, in the Al_x_Ga_1−x_N-on-Sapphire architecture considered in the following, an x = 0.65 provides a sufficiently high index contrast, transparency down to 260 nm, SBS-activity, negligible TPA effect (cut-off at 486 nm) and fabrication relaxed dimensions.

Moreover, considering that the growth of AlN on sapphire includes a *θ* = 30° rotation of AlN a-axis around the growth direction (c-axis, see Fig. 1(c)), it results a lattice mismatch of 13.3%^28^. Thus, similar rotation in case of an Al_x_Ga_1−x_N waveguide on sapphire substrate can be assumed. Furthermore, Sellmeier’s index equations for AlN and Al_x_Ga_1−x_N^28^, sapphire and alumina^26^ have been used in the following simulations to take into account the index dispersion of the material.

By referring to Eq. (4), knowledge of the acoustic decay parameter *α* is required to obtain numerical values to be compared with experimental results. Generally speaking, this parameter can be derived from the dynamic viscosity tensor $\bar{{\boldsymbol{\eta }}}$^23^. It this context, we have tested our software code using the Ge platform proposed in^24^. We found that our *α* estimation presents a relative change <3% if compared with the numerical results obtained in^24^. We guess that this weak difference depends exclusively on the mesh used to perform the calculations, confirming the robustness of our implementations. Since at the best of our knowledge $\bar{{\boldsymbol{\eta }}}$ is not available in the literature for both AlN and GaN, we cannot use Eq. (8) to estimate the alloy phonon viscosity tensor. At this step since the goal of this work is to demonstrate the feasibility of SBS effect in the Al_x_Ga_1−x_N platform, we briefly discuss the different way to estimate the acoustic decay parameter. In absence of the viscosity tensor elements, a possible approach could be to consider the Al_x_Ga_1−x_N alloy as an isotropic material and then estimate the phonon viscosity coefficient by means of approaches based on the fluctuation dissipation theorem, phonon hydrodynamics and kinetic theory. However, we believe that the isotropic assumption is too strong for wurtzite crystals. Moreover, the above-mentioned approaches applied to the silicon gave results which differ from each other for order of magnitude, and very far from the experimental values^29^. In this context, we can conclude that these approaches are not suitable to obtain a consistent estimation of the *α* parameter for Al_x_Ga_1−x_N platform. In this sense, we believe that the use of the acoustic quality factor can represent an efficient choice to demonstrate the range of feasibility of the SBS effect in Al_x_Ga_1−x_N. Therefore, in the following, we have estimated the decay parameter as *α* = *Ω*_*B*_*E*_*ac*_/(2*Q*_*factor*_*P*_*ac*_), where *Q*_*factor*_ represents the acoustic quality factor, considered as a numerical parameter in the following investigations. The terms *E*_*ac*_ and *P*_*ac*_ represent the acoustic mode energy and power, respectively^23^. Additionally, experimental measurements of $\bar{{\boldsymbol{\eta }}}$ could be used to better set the values of *α* in order to improve the model predictions of the BSBS effect in the Al_x_Ga_1−x_N platform operating in the visible and UV-vis ranges.

Note that in our simulations the outer boundary of the calculation domain is set to be significantly larger than the waveguide core to avoid any influence on the modal properties of the acoustic waves. Consequently, the solution of the problem in Eq. (3) leads to an evaluation of all possible acoustic modes with both real and non-real values of *Ω*_*B*_. The latter corresponds to leaky modes, which are strongly confined due to the strong reflection of the acoustic wave at the core-substrate (cover) interfaces, but which are capable of dissipating energy away from the waveguides. As well detailed in^30^, the use of the eigenvalue problem of Eq. (3) represents an efficient tool to study the SBS interaction. Indeed, the imaginary part of *Ω*_*B*_ leads to an estimation of the rate (1/*τ*) of acoustic energy lost by the core into the substrate (cover) due to mode leakage as: 1/*τ* = *Im*(*Ω*_*B*_)^30^. It is evident, thus, that the acoustic mode lifetime is strongly dependent on both the material distribution and the waveguide cross section. However, Eq. (11) represents an efficient method to select the material distribution for the existence of guided acoustic modes.

Although the SBS effect has been demonstrated also for radiative and leaky acoustic modes, in the following we will analyze only the technological platforms for which Eq. (11) is satisfied. In this context for example, both Sapphire/Al_0.65_Ga_0.35_N/Air and Sapphire/GaN/Air platforms operating at 450 nm and 780 nm, respectively, record the parameter *Im*(*Ω*_*B*_) in the range [10^−6^ to 10^−5^] (numerical zero). As result, the only SBS-active acoustic modes are guided modes, and then the limiting effect on the mechanical quality factor is represented by the dynamic viscosity of the materials. Therefore as a starting point for our investigation, we assume a total mechanical quality factor *Q*_*factor*_ = 3000, driven by the fact that this value has been demonstrated to be consistent with the dynamic viscosity effects in non-suspended waveguides^24^. On the other hand, the mechanical quality factor does not influence the above-discussed acoustic mode behaviour but only the BSBS power gain, whose changes are shown in the following Table 3 for different AlGaN-based technology platforms and quality factors in the range from 1500 to 3000.

**Table 3:** BSBS parameters for different materials.

Technological platform	Pump wavelength [nm]	Power gain [W^−1^m^−1^]	Stokes Shift [GHz]
Air Suspended Si; ref.^22^	1550	890	~13.5
Ge [100] embedded in Si_3_N_4_; ref.^24^	4000	500	~6.5
Ge [110] embedded in Si_3_N_4_; ref.^24^	4000	~1000	~6.5
As_2_S_3_ on silica; ref.^5^	1544.77	321.74	~7.6
Sapphire/Al_0.65_Ga_0.35_N/Alumina (this work) *Q*_*factor*_ [1500–3000]	450	275 ÷ 556	52.56
Sapphire/Al_0.65_Ga_0.35_N/Air (this work) *Q*_*factor*_ [1500–3000]	450	823 ÷ 1647	49.86
Sapphire/AlGaN/Air (this work) *Q*_*factor*_ [1500–3000]	780	640 ÷ 1280	23.3

**Figure 2:**
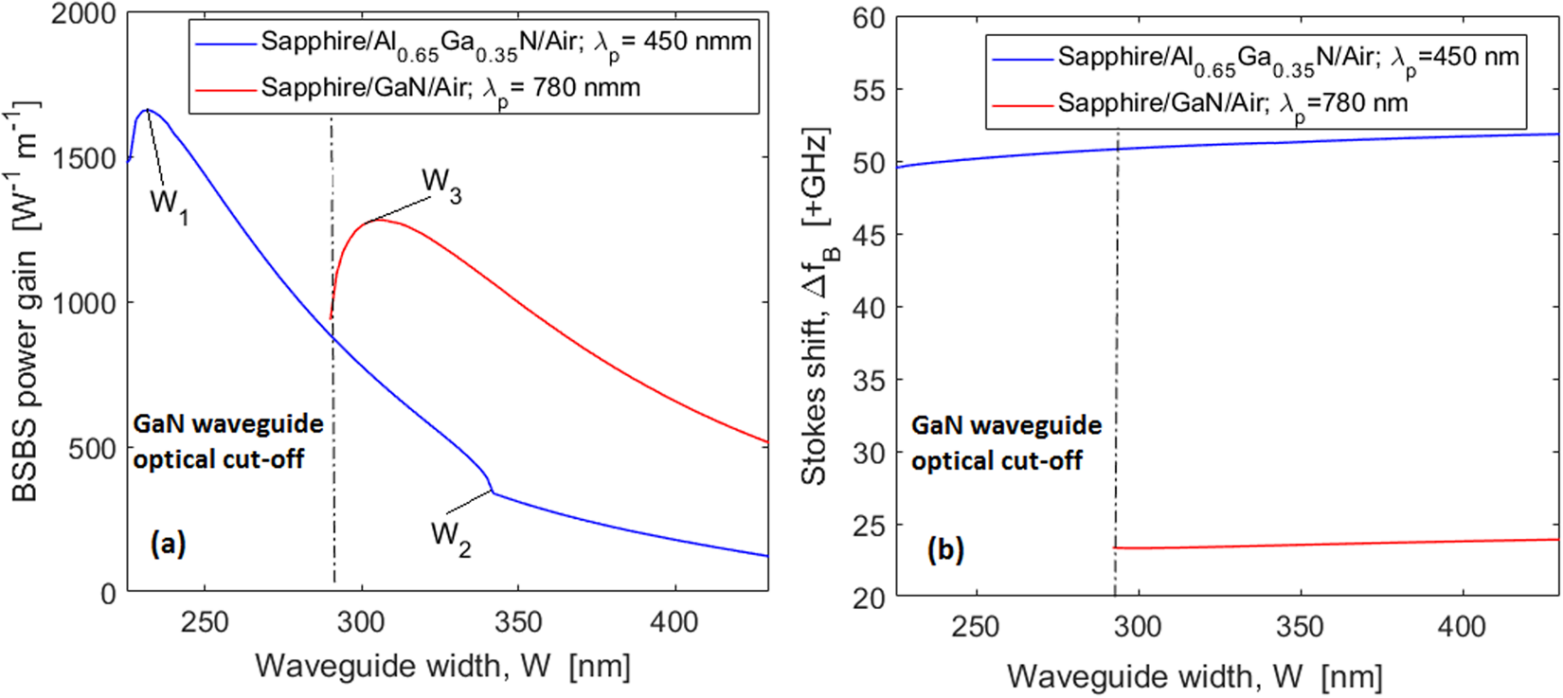
(**a**) BSBS power gain for an AlGaN waveguide as a function of waveguide width, for both Sapphire/Al_0.65_Ga_0.35_N/Air (at *λ*_*p*_ = 450 nm) and Sapphire/GaN/Air (at *λ*_*p*_ = 780 nm) platforms; (**b**) Stokes shift as a function of waveguide width, for both Sapphire/Al_0.65_Ga_0.35_N/Air (at *λ*_*p*_ = 450 nm) and Sapphire/GaN/Air (at *λ*_*p*_ = 780 nm) platforms. The waveguide height is *H* = 300.

It is interesting to compare the Sapphire/Al_0.65_Ga_0.35_N/Air and Sapphire/GaN/Air platforms, given the non-ideality effects such as two photon absorption (TPA) and free carrier absorption. To avoid TPA, the pump wavelength should be chosen to be close to the TPA cut-off wavelength. Figure 2 (a) and (b) show the BSBS power gain and the Stokes shift (${\rm{\Delta }}{f}_{B}={{\rm{\Omega }}}_{B}/2\pi$) versus the waveguide width for both Sapphire/Al_0.65_Ga_0.35_N/Air and Sapphire/GaN/Air platforms operating at 450 nm and 780 nm, respectively. The numerical calculations have been carried out as described in the Method section, assuming *H* = 300 nm, and the acoustic quality factor, *Q*_*factor*_ = 3000.

**Figure 3:**
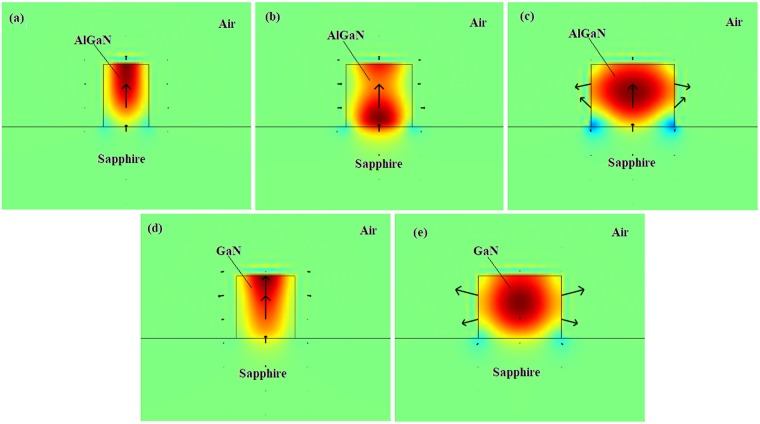
Color-maps of the longitudinal component of the mechanical displacement. Arrows indicate the in-plane acoustic displacement, assuming *H* = 300 nm; (**a**) Sapphire/Al_0.65_Ga_0.35_N/Air, *W* = 220*nm* < *W*_1_; (**b**) Sapphire/Al_0.65_Ga_0.35_N/Air, *W*_1_ < *W* = 320*nm* < W_2_; (**c**) Sapphire/Al_0.65_Ga_0.35_N/Air, *W* = 400 *nm* > *W*_2_; (**d**) Sapphire/GaN/Air, *W* = 280 *nm* < *W*_3_; (**e**) Sapphire/GaN/Air, *W* = 400 *nm* > *W*_3_. The pump laser wavelengths are 450 nm and 780 nm for Sapphire/Al_0.65_Ga_0.35_N/Air and Sapphire/GaN/Air, respectively.

Our best results are relevant to the case Sapphire/Al_0.65_Ga_0.35_N/Air, where we have recorded a maximum (and especially high) power gain of 1647 W^−1^m^−1^ and a Stokes shift of 49.86 GHz, for *H* = 300 nm, *W*~236 nm. Likewise, the maximum power gain obtained for the Sapphire/GaN/Air platforms operating at 780 nm is 1280 W^−1^m^−1^ with *H* = 300 nm, *W* = 306 nm. Although the BSBS power gain for Sapphire/GaN/Air platform is comparable with that of the Sapphire/Al_0.65_Ga_0.35_N/Air structure, the Stokes shift suffers from 46.7% reduction, as shown in Fig. 2(b). In the plot of Fig. 2(a) it is possible to find characteristic values for the waveguide width, named as *W*_1_, and*W*_2_, for Sapphire/Al_0.65_Ga_0.35_N/Air and *W*_3_ for Sapphire/GaN/Air. These values correspond to different mechanical deformations induced by the acoustic mode generating the Brillouin gain. In particular in the case of Sapphire/Al_0.65_Ga_0.35_N/Air platform, we record that for *W* < *W*_1_, the in-plane acoustic displacement (${u}_{T}=\sqrt{{|{u}_{x}|}^{2}+{|{u}_{z}|}^{2}}$) is dominant in the vertical direction with the peak of the longitudinal component of the mechanical displacement close to the superior waveguide edge. This peak shift toward the middle of the waveguide cross section, for *W* = *W*_1_. Increasing the *W* value, *u*_*T*_ remains exclusively oriented in the vertical directions until the condition *W* = *W*_2_ is reached. For *W* > *W*_2_, the in-plane displacement is mainly characterized by a horizontal expansion close to the lateral waveguide sidewalls. Finally, in the case of Sapphire/GaN/Air platform, the waveguide width *W*_3_ separates the region in which *u*_*T*_ is mainly oriented in the vertical (*W* > *W*_3_) or horizontal direction (*W* > *W*_3_). The previous discussion is clearly shown in the panels of Fig. (3), where the color-maps for the longitudinal component of the mechanical displacement is plotted for different values of the waveguide width (*W*), assuming *H* = 300 nm, and including the arrows for the in-plane acoustic displacement *u*_*T*_.

**Figure 4:**
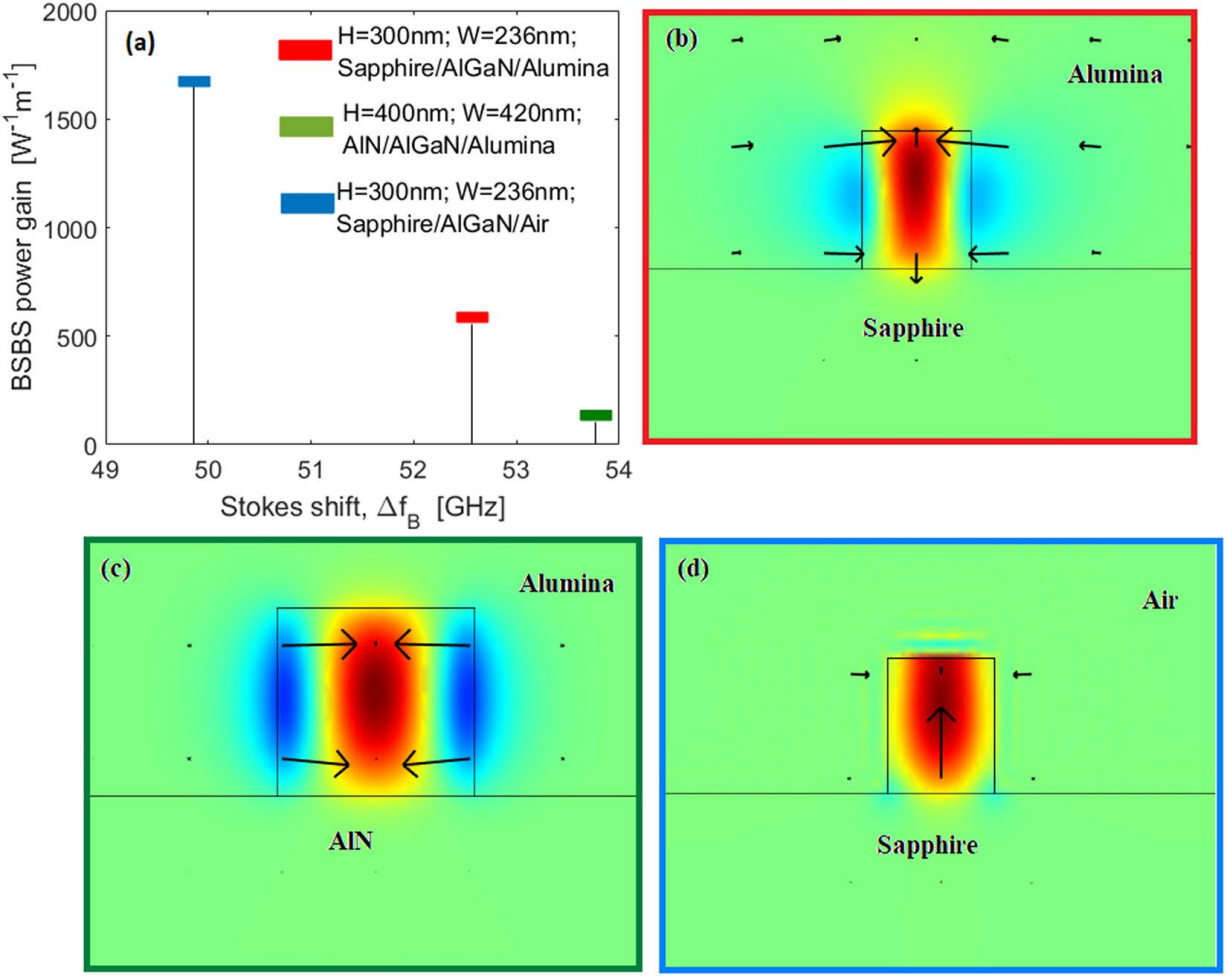
(**a**) Calculated BSBS features (Stokes shift and power gain), assuming *Q*_*factor*_ = 3000, and *λ*_*p*_ = 450 nm. Red, green, and blue bars represent BSBS gain for three different waveguide compositions: Sapphire/AlGaN/Alumina, AlN/AlGaN/Alumina, and Sapphire/AlGaN/Air. (**b**) Longitudinal component of the mechanical displacement for Sapphire/AlGaN/Alumina; (**c**) same for AlN/AlGaN/Alumina. (**d**) same for Sapphire/AlGaN/Air.

Since experimental investigations have not been presented in literature for the BSBS effect in Al_x_Ga_1−x_N waveguides, we propose in Fig. 4 a comparison between waveguide structures with different Substrate/Al_0.65_GaN_0.35_/Cover compositions. In particular, in Fig. 4(a) the BSBS power gain and the relevant Stokes shift are shown as a bar plot for three different material compositions: Sapphire/Al_0.65_GaN_0.35_/Alumina (red bar), AlN/Al_0.65_GaN_0.35_/Alumina (green bar), and Sapphire/Al_0.65_GaN_0.35_/Air (blue bar), assuming *λ*_*p*_ = 450 nm and *Q*_*factor*_ = 3000. For each case, the waveguide cross sections have been selected in order to maximize the power gain. The color-map panels (Fig. 4(b–d)) show the longitudinal component of the mechanical displacement, the arrows representing the in-plane acoustic displacement *u*_*T*_.

The data of Fig. (4) indicate that the sapphire substrate leads to higher power gain than the AlN substrate material, although it suffers from a larger lattice mismatch with the AlGaN layer. To the best of our knowledge, theoretical or experimental values have not been proposed in the literature for the dynamic tensor $\bar{{\boldsymbol{\eta }}}$. Thus, in a first approximation, it seems reasonable to assume the *Q*_*factor*_ ranging from 1500 to 3000. In this context, we can suppose that the BSBS power gain in the Sapphire/AlGaN/Air platform operating in the visible could change from 823 W^−1^m^−1^ to 1647 W^−1^m^−1^.

We believe that the Sapphire/AlGaN/Air platform could be considered the best trade-off between high power gain and fabrication constraints. Indeed, this waveguide cross section removes the difficult fabrication conditions needed for the suspended waveguides. It is important to compare our results with those obtained with different technological platforms. An immediate comparison can be made with the silicon waveguide operating in the near-infrared region^22^. Recently, waveguides based on germanium embedded in Si_3_N_4_ have been proposed in order to induce the BSBS effect in photonic integrated circuits operating in the mid-IR spectrum^24^. At the same time, the chalcogenide As_2_S_3_^5^ has attracted a lot of attention as a platform to induce the BSBS effect in an integrated structure, mainly because it has a high acousto-optic overlap, does not suffer from any parasitic losses such as two photon absorption (typical in the silicon platform), and can support waveguides with losses below 0.5 dB/cm. In Table 3 the BSBS parameters are summarized for the platforms above mentioned.

Although the numerical results for the case of a perfect crystal indicate that the platform Al_x_Ga_1−x_N on Sapphire should be a good candidate to realise high backward Brillouin effect in the visible range, the lattice mismatch-induced defects could represent a significant detrimental effect for BSBS effects, increasing the optical propagation loss of the strip waveguide and influencing the BSBS power gain.

**Figure 5:**
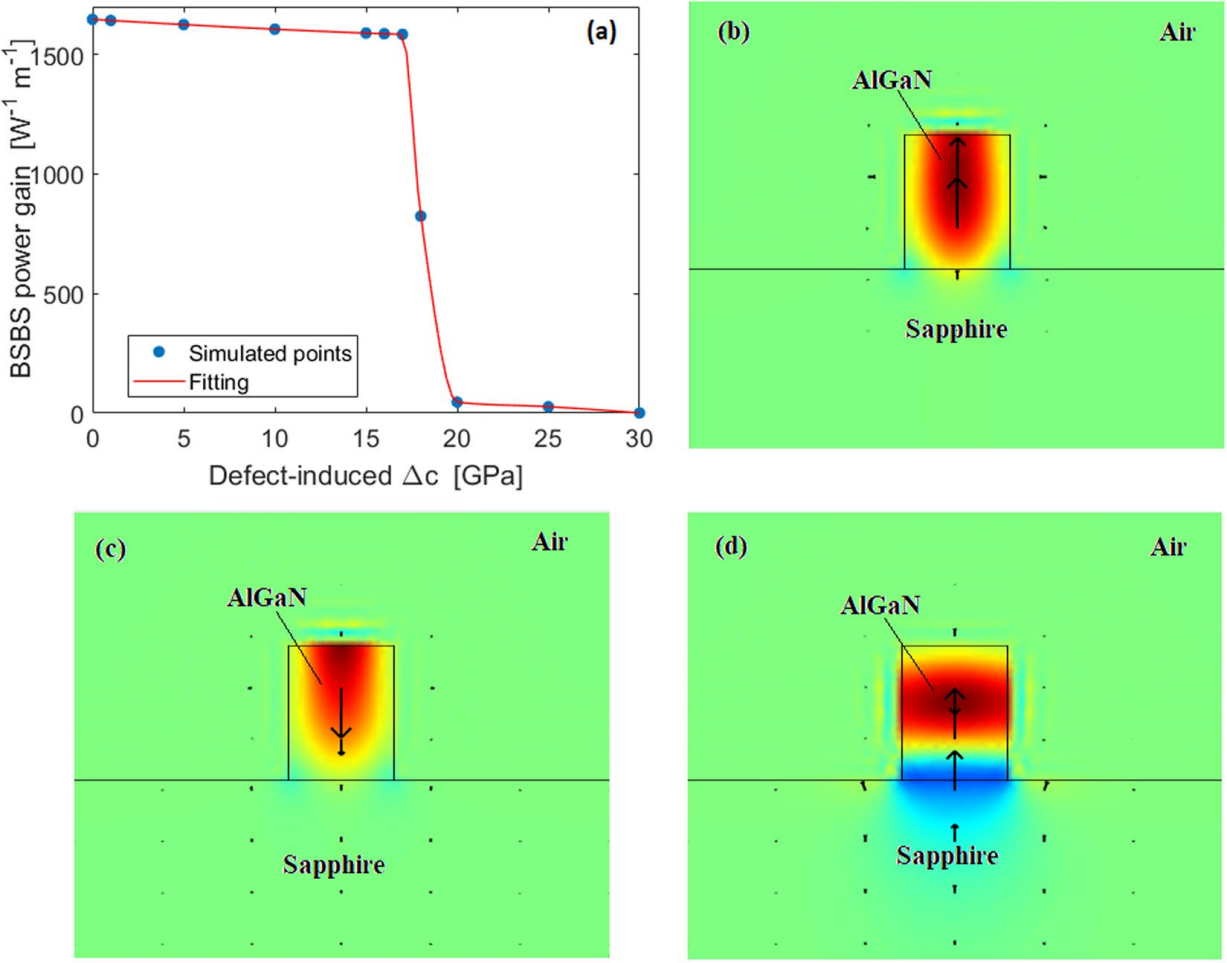
(**a**) BSBS power gain as a function of the defect-induced Δ*c*, assuming: *H* = 300 nm, *W* = 236 nm, *λ*_*p*_ = 450 nm, *Q*_*factor*_ = 3000 and air cover; (**b**) Longitudinal component of the mechanical displacement for Sapphire/Al_0.65_Ga_0.35_N/air with Δ*c* = 0 GPa (**c**) same for Δ*c* = 18 GPa; (**d**) same for Δ*c* = 22 GPa.

However, it has been rarely studied how the point defect changes the mechanical properties. Only recently it was found that defects such as uniformly distributed vacancies or vacancy clusters induce, in hexagonal systems, an increase in *c*′_11_, *c*′_33_, and *c*′_44_ and a decrease of *c*′_12_, and *c*′_13_^31^. Due to the lack of experimental data for the alloy Al_x_Ga_1−x_N, we guess, as hypothesis of the worst case, that all five independent stiffness tensor elements suffer from the same change, ±Δ*c*. In this context, according to the conclusions presented in^29^, we assume $\bar{c}{^{\prime} }_{11}=c{^{\prime} }_{11}+{\rm{\Delta }}c$, $\bar{c}{^{\prime} }_{33}=c{^{\prime} }_{33}+{\rm{\Delta }}c$, $\bar{c}{^{\prime} }_{44}=c{^{\prime} }_{44}+{\rm{\Delta }}c$, $\bar{c}{^{\prime} }_{12}=c{^{\prime} }_{12}-{\rm{\Delta }}c$, and $\bar{c}{^{\prime} }_{13}=c{^{\prime} }_{13}-{\rm{\Delta }}c$, where Δ*c* is the defect-induced stiffness change, and *c*′_*ij*_ is the stiffness tensor element for the perfect crystal (listed in Table 1). In Fig. 5(a), the BSBS power gain is shown as a function of the defect-induced stiffness change, Δ*c*, assuming air cover, *H* = 300 nm, *W* = 236 nm, *λ*_*p*_ = 450 nm and *Q*_*factor*_ = 3000. Our investigations indicate that with the increase of Δ*c* from 0 to 30 GPa, the bulk modulus (defined as in^31^) decreases monotonically, inducing the reduction of Al_x_Ga_1−x_N resistance to uniform compression. At the same time, shear modulus, and Young’s modulus (defined as in^31^) increase monotonically, while the Poisson’s ratio decreases. This means that the presence of defects could enhance the resistance to shear and uniaxial stresses. Thus, the curve shape of Fig. 5(a) can be justified by arguing that the defect-induced Δ*c* does not produce any material degeneration but rather induces a reduction in the acoustic mode confinement (see Fig. 5(b,c)) or a change in the mechanical displacement spatial distribution (see Fig. 5(d)). Indeed, for Δ*c* ≥ 20 GPa, the platform Al_x_Ga_1−x_N on Sapphire become weakly Brillouin active, showing a BSBS power gain less than 46 W^−1^m^−1^. However, a BSBS power gain reduction of about 4% can be obtained if the defect-induced Δ*c* is kept below 17 GPa. In this condition, the maximum changes in the bulk, shear, Young’s moduli and Poisson’s ratio are 4.7%, 14%, 10.4%, and 15.2%, respectively. Experimental measurements could be used to realise a relationship between the defect-induced Δ*c* and the density of the defects. Thus, the epitaxial growing of Al_0.65_Ga_0.35_N on the sapphire can be used if the mechanical properties of the defective Al_0.65_Ga_0.35_N changes in the limits above mentioned. However, considering that Al_0.65_Ga_0.35_N has a thermal coefficient of expansion close to that of silicon and silicon has been successfully bonded to sapphire, we could speculate that low-defect Al_0.65_Ga_0.35_N can be bond directly on Sapphire. Alternatively, the bonding could be performed using an ultrathin inter-layer of SiO_2_ on the sapphire.

In conclusion, we believe that the numerical results presented here show that the technological platform based on Al_x_Ga_1−x_N, with its very high gain and very high microwave-frequency shift, could be suitable to realise on chip devices based on the Brillouin scattering effect and operating in the visible wavelength range, such as Brillouin ring lasers and photonic microwave sources.

### Forward SBS

**Figure 6:**
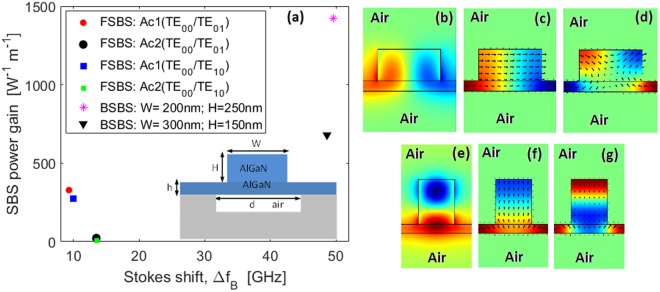
(**a**) Calculated inter-modal FSBS and BSBS features (Stokes shift and power gain) at 450 nm wavelength. Red and black circles represent the FSBS (TE_00_ → TE_01_; *H* = 250 nm; *h* = 50 nm; *W* = 200 nm, *d* = 400 nm) power gain for the acoustic waves Ac1, and Ac2, respectively. Blue and green squares represent the FSBS (TE_00_ → TE_10_; *H* = 150 nm; *h* = 50 nm; *W* = 300 nm, *d* = 450 nm) power gain for the acoustic waves Ac1, and Ac2, respectively. The asterisk and triangle represent the maximum BSBS power gain for the considered waveguides. (**b**–**e**) x-component Electric field of the TE_10_ − TE_01_ Stokes mode distributions. (**c**,**d**) Longitudinal component of the mechanical displacement induced by (TE_00_ → TE_10_), and for Ac1-Ac2. (**f**,**g**) Longitudinal component of the mechanical displacement induced by (TE_00_ → TE_01_), and for Ac1-Ac2.

Forward SBS (FSBS) is generally weaker that the BSBS in large core size waveguides. On the contrary, integrated waveguides with nanoscale sizes can have large FSBS gain due to their strong acoustic mode confinement. The unique feature of the FSBS process is that the pump-Stokes coupling can involve optical modes with different symmetry and polarization^23^. In the following, we investigate the possibility of inducing inter-modal FSBS power gain in the Al_x_Ga_1−x_N platform operating in the visible spectrum range. We assume that the pump light, (${\tilde{{\boldsymbol{e}}}}_{p}$, *β*_*p*_(*ω*_*p*_)), guided in the symmetric TE_00_ mode, scatters to a red-shifted wave (${\tilde{{\boldsymbol{e}}}}_{S}$, *β*_*S*_(*ω*_*p*_ − *Ω*)) that is guided in the anti-symmetric Stokes TE_10_ or TE_01_ mode. Our preliminary investigations indicate that elastic waves are FSBS-active only for the suspended waveguide, as sketched in the inset of Fig. 6(a). Moreover, suspended waveguides can induce high BSBS power gain with the advantage to avoid the detrimental effect of the acoustic decay contribution inside the substrate. In this sense, we have considered two Brillouin-active waveguides: *H* = 150 nm; *h* = 50 nm; *W* = 300 nm, *d* = 450 nm for the energy transfer between TE_00_ → TE_10_; and *H* = 250 nm; *h* = 50 nm; *W* = 200 nm, *d* = 400 nm for the energy transfer between TE_00_ → TE_01_.

It is worth noting that the considered waveguides support numerous guided elastic waves, but only a small number of them are Brillouin-active. Figure 6(a) shows the FSBS inter-modal power gain and the Stokes shift for two FSBS-active acoustic waves (Ac1 and Ac2). In the simulations, we have assumed *q* ≅ *β*_*p*_(*TE*_00_) − *β*_*p*_(*TE*_10(01)_), *λ*_*p*_ = 450 nm and *Q*_*factor*_ = 3000. The data indicate that it is possible to obtain a FSBS inter-modal power gain of about 282 W^−1^m^−1^ and 328.56 W^−1^m^−1^ for the coupling Ac1- (TE_00_ → TE_10_) and Ac1-(TE_00_ → TE_01_), respectively. In the same condition, the Stokes shift changes from 10 GHz to 9.3 GHz. Moreover, the acoustic wave Ac2 induces a weak power gain of 6.45 W^−1^m^−1^ and 23.2 W^−1^m^−1^ for the (TE_00_ → TE_10_) and (TE_00_ → TE_01_) mechanism, respectively. For comparison, we have reported in Fig. 6(a) the maximum BSBS power gain for both the considered waveguides. The simulations show a BSBS power gain of 1424.5 W^−1^m^−1^ at Δ*f*_*B*_ = 49.55 GHz and 679.4 W^−1^m^−1^ at Δ*f*_*B*_ = 48.63 GHz for *W* = 200 nm, *H* = 250 nm, and *W* = 300 nm, *H* = 150 nm, respectively.

It is worth noting that the character of the Brillouin-active acoustic waves changes significantly, depending on the spatial distribution of the Stokes mode (see Fig. 6(b), and Fig. 6(e)) involved in the scattering process. In particular, the coupling condition Ac1- (TE_00_ → TE_10_) induces a weak flexural character with a transverse mechanical displacement (see Fig. 6(c)). Differently, a flexural character with vertical in plane mechanical displacement (see Fig. 6(f)) is induced by Ac1- (TE_00_ → TE_01_). Similarly, we have considered two Brillouin-active GaN suspended waveguides at 780 nm wavelength: *H* = 200 nm; *h* = 50 nm; *W* = 450 nm, *d* = 675 nm for the energy transfer between TE_00_ → TE_10_; and *H* = 470 nm; *h* = 50 nm; *W* = 346 nm, *d* = 692 nm for the energy transfer between TE_00_ → TE_01_. The simulations, not shown here, indicate the FSBS inter-modal power gain and Stokes shift are as low as 88.68 W^−1^m^−1^ and 5.99 GHz, 72.92 W^−1^m^−1^, and 4.53 GHz for the coupling Ac1- (TE_00_ → TE_10_) and for Ac1-(TE_00_ → TE_01_), respectively.

### Racetrack Brillouin Laser

**Figure 7:**
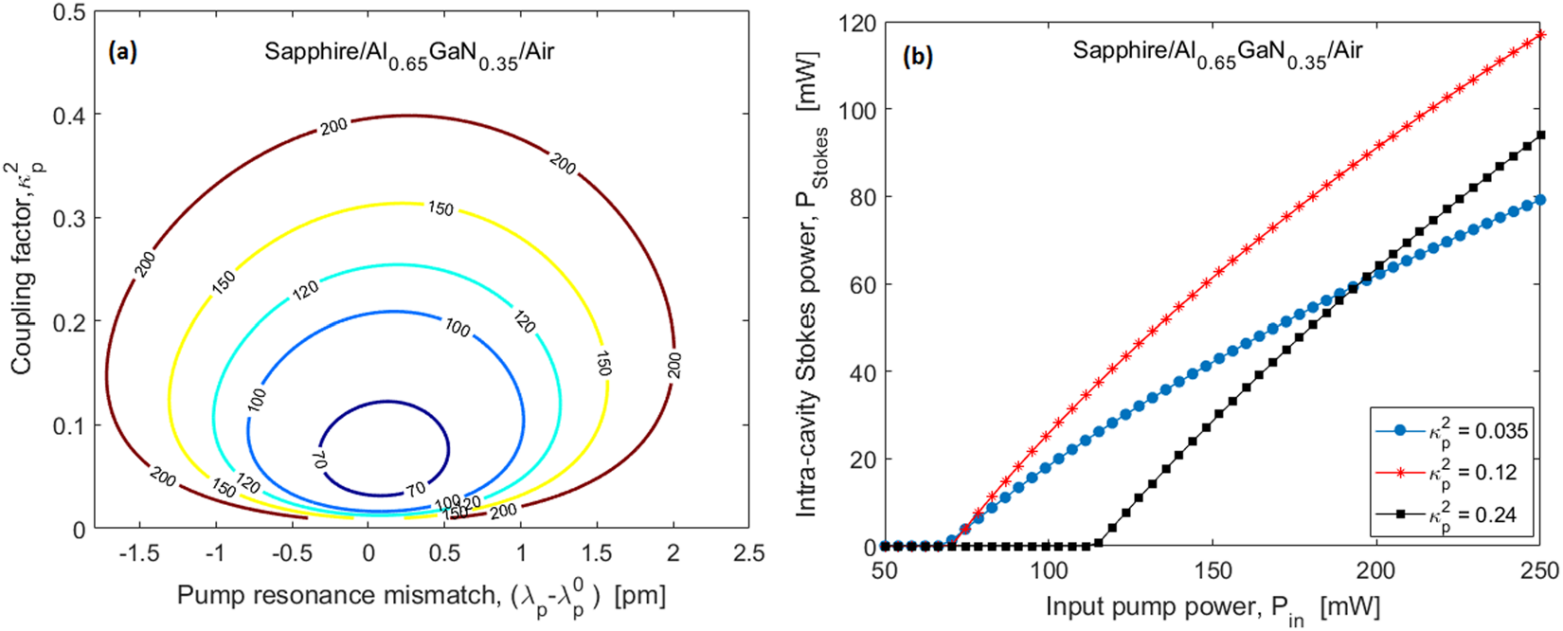
(**a**) Threshold power as a function of the pump resonance mismatch and pump coupling factor; (**b**) Intra-cavity Stokes power as a function of the input pump power for different values of the pump coupling. Waveguide cross-section: *H* = 300 nm, *W* = 236 nm, pump wavelength around 450 nm, and acoustic quality factor *Q*_*factor*_ ~3000.

Recently, the on-chip Brillouin lasers have been the subject of numerous research efforts^16,19,32–35^. In this context we investigate the BSBS lasing as it occurs in racetrack resonators based on Sapphire/Al_0.65_GaN_0.35_/Air waveguides at the visible wavelength of 450 nm. In particular, we consider the architecture sketched in^18^ where the input pump signal, *S*_*p*_ (${P}_{in}={|{S}_{p}|}^{2}$), is injected into the racetrack resonator by means of the evanescent coupling between the resonant microcavity and the input external bus. According to the mathematical model proposed in our previous work^18^, the calculations at *λ*_*p*_ = 450 nm give a nonlinear Kerr effective modal area (defined as in^36^) *A*^*kerr*^ = 5.4 × 10^−2^ μm^2^ and the group effective index, *n*_*g*,*p*_ = *n*_*g*,*s*_ = 2.7130. The conventional approach for designing a Brillouin laser based on optical resonators consists in satisfying the condition Δ*f*_*FSR*_ = Δ*f*_*B*_, whereΔ*f*_*FSR*_ is the free spectral range (FSR) of the racetrack resonator. Consequently, by imposing that the pump and Stokes frequencies coincide with two adjacent cavity resonances, we obtain that the first-order estimation for the cavity length (${L}_{cav}^{0}$) is 2.2177 mm. In this context^18^, the Brillouin laser threshold (${P}_{in}^{(th)}={|{S}_{p}^{(th)}|}^{2}$) is shown in Fig. 7(a) as a function of the pump and Stokes coupling factors (${\kappa }_{p}^{2}={\kappa }_{S}^{2}$), and the pump resonance mismatch, $({\lambda }_{p}-{\lambda }_{p}^{0})=-\,2\pi {c}_{0}({\omega }_{p}-{\omega }_{p}^{0})/{\omega }_{p}^{2}$, where ${\omega }_{p}^{0}\,$is the pump resonance angular frequency. In the simulations, we have assumed the linear loss coefficient, α_loss_ = 5dB/cm, and the Kerr nonlinear refractive index,*n*_2_ = 3 × 10^−19^ m^2^/W^37^. The curves in Fig. 7(a) show that locking the pump to the cavity resonance, a minimum threshold power ${P}_{in,min}^{(th)}$ of about 70 mW can be reached when setting the pump coupling factor to ${\kappa }_{p}^{2}=0.21$. Moreover, similar investigations show that ${P}_{in,min}^{(th)}$ ranges from 25 mW (${\kappa }_{p}^{2}=0.07$) to 250 mW (${\kappa }_{p}^{2}=0.17$), when changing *α*_*loss*_ from 3 dB/cm to 10 dB/cm. It is worth pointing out that the minimum Brillouin laser thresholds obtained in these simulations are lower than those shown in^18^, as a result of the higher Stokes shift produced by the Sapphire/Al_0.65_GaN_0.35_/Air platform. This results in a reduced cavity length (~5 times less) and, then, an increase of the resonator enhancement.

**Figure 8:**
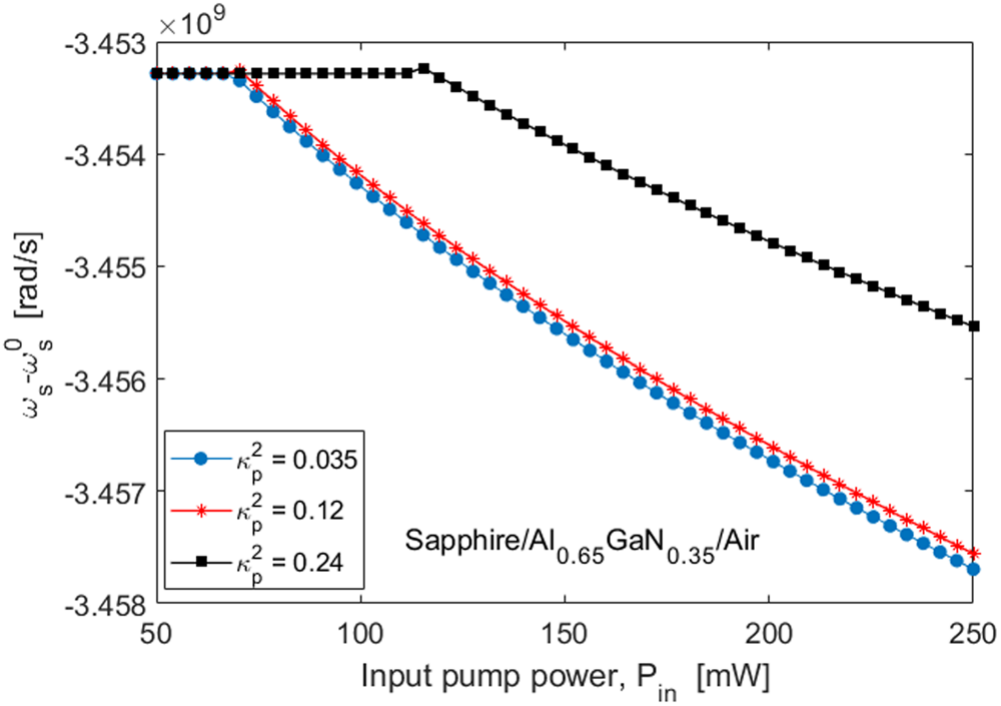
Mismatch between the lasing frequency and the resonance cavity frequency as a function of the input pump power for different values of the pump coupling factor. Waveguide cross-section: *H* = 300 nm, *W* = 236 nm, pump wavelength around 450 nm, and acoustic quality factor *Q*_*factor*_ ~3000.

As detailed in^18^, the use of ${L}_{cav}^{0}$ does not allow any pushing and pulling effects occurring in the Brillouin lasing process to be compensated. A better approach consists in particularizing the cavity length *L*_*cav*_ to realize a resonator FSR that is able to fully compensate the pushing effect induced by the pump-Stokes cross-phase modulation. Thus, our simulations indicate that the difference (${L}_{cav}-{L}_{cav}^{0}$) increases by increasing the coupling factor with slope 0.69 µm/%. In this context, Fig. 7(b) shows the intra-cavity Stokes power as a function of the input pump power (*P*_*in*_), for different values of the pump coupling factor ${\kappa }_{p}^{2}$. Although the Brillouin lasers with ${\kappa }_{p}^{2}=0.035$ and ${\kappa }_{p}^{2}=0.12$ are characterized by the same threshold powers (see Fig. 7(a)), they also exhibit different Stokes emission powers as a result of a different external efficiency. Moreover, the three different Brillouin lasers considered Fig. 7(b) are characterized by a coupling factor κ^2^ = 0.035, 0.12 and 0.24, a cavity length *L*_*cav*_ = 2.228, 2.234, and 2.242 mm, ${\omega }_{p}^{0}=4.1887\times {10}^{15}$, 4.1888 × 10^15^ and 4.1888 × 10^15^ rad/s, and ${\omega }_{s}^{0}=4.1883\times {10}^{15}$, 4.1885 × 10^15^ and 4.1885 × 10^15^ rad/s. Under these conditions, the Stokes lasing frequency is shown in Fig. 8, in which the Stokes-SPM pushing effect manifests itself as a decreasing of the emission frequency by increasing the input pump power.

Moreover, we estimate the influence of thermal effects on the laser operation. A temperature change (Δ*T* = *T* − *T*_0_) with respect to the reference (*T*_0_, i.e. room temperature), induces a shift of resonance angular frequencies as ${\omega }_{i}^{0}(T)-{\omega }_{i}^{0}({T}_{0})={\omega }_{i}^{0}({T}_{0})({\alpha }_{therm}+(1/n)(dn/dT))$ where *α*_*therm*_ = 4.535 × 10^−6^ K^−1^ and (*dn*/*dT*) = 7.94 × 10-5 K^−1^ are the Al_0.65_Ga_0.35_N thermal expansion and thermo-optic coefficients, respectively, and estimated by means of Eq. (8), assuming *α*_*therm*_(*AlN*) = 5.26 × 10^−6^ K^−1^ *α*_*therm*_(*GaN*) = 3.17 × 10^−6^ K^−1^, *dn*/*dT*(*AlN*) = 3.6 × 10^−5^ K^−1^, and *dn*/*dT*(*GaN*) = 1.6 × 10^−4^ K^−1 ^^38^. Thus, assuming the temperature change of the Stokes frequency shift as a function of (1/*Ω*_*B*_)(*dΩ*_*B*_/*dT*), and the locking of the pump laser to the cavity resonance, we obtain the following quadratic law:

12 $${\omega }_{S}(T)-{\omega }_{S}^{0}(T)={a}_{T}{\rm{\Delta }}{T}^{2}+{b}_{T}{\rm{\Delta }}T+{c}_{T}$$

where a_T_, b_T_, and c_T_are fitting coefficients, namely 5.6 × 10^4^ rad K^−2^/s, 1.8 × 10^5^ rad K^−1^/s, and −8.863 × 10^9^ rad/s, for κ^2^ = 0.12.

It is worth to outling that, the results of this sub-section should not be considered as a rigorous design of the Brillouin laser on the AlGaN platform. Indeed, due to the lack of values on the phonon viscosity tensor, we have uncertainity on the BSBS power gain values. On the contrary, the results above proposed should be seen a case of study in order to demonstrate the feasibility of the Brullouin laser on the AlGaN platform and operating in the visible range, in which the main physical effects and the design rules have been evidenced.

## Conclusions

In this paper, mathematical modeling based on a multiphysics physical approach has been implemented to investigate the BSBS and FSBS scattering in the visible spectrum range. The simulations have been performed including the electric-photoelastic, magnetic-photoelastic and material interface displacement effects in order to obtain a consistent estimation of the Brillouin-active acoustic modes, Brillouin power gain and Stokes frequency shift. Through the study of Sapphire/Al_0.65_GaN_0.35_/Alumina, and Sapphire/Al_0.65_GaN_0.35_/Air waveguides, we have demonstrated that the BSBS effect can be significantly induced in nanoscale structures at 450 nm pumping. The predicted power gains for backward SBS ranged from roughly 556 W^−1^m^−1^ for a waveguide of Sapphire/Al_0.65_GaN_0.35_/Alumina to roughly 1647 W^−1^m^−1^ for Sapphire/Al_0.65_GaN_0.35_/Air, with Stokes shifts changing from 52.56 GHz to 49.86 GHz. The BSBS power gain could suffer from a reduction of 4% if the defect-induced stiffness change is kept below 17 GPa. Moreover, our investigations have been applied to the case of Al_0.65_GaN_0.35_ rib waveguide with air pocket in SiO_2_, demonstrating that Al_0.65_GaN_0.35_ suspended waveguides are suitable to induce both inter-modal coupling FSBS and BSBS power. Our analysis and simulations have recorded a FSBS inter-modal power gain of about 271.94 W^−1^m^−1^ and 328.56 W^−1^m^−1^ for the coupling (TE_00_ → TE_10_) and (TE_00_ → TE_01_), respectively. In the same coupling condition, the Stokes shift changed from 10 GHz to 9.3 GHz. Moreover, our simulations have shown a BSBS power gain and a Stokes shift of 1421.5 W^−1^m^−1^, and 49.55 GHz, respectively. From these results, the AlGaN technological platform can be considered as a very good candidate for nonlinear Brillouin applications since it can simultaneously guarantee both large power gains and large Stokes frequency shifts. Among different applications, we have theoretically demonstrated the possibility of realizing a practical on-chip waveguided visible Brillouin-laser source based on the BSBS effect induced inside an integrated racetrack resonator.

## Methods

Our procedure for the calculations of both power gain and Stokes frequency shift is based on the model presented in the Theory Section and is described in more details below: With the aim of realizing self-consistent simulations, we have implemented an integrated algorithmic procedure based on home-made code and commercial software using the full-vectorial Finite Element Method (FEM)^39^. In particular, for a given waveguide cross-section, the FEM approach is also used to solve the Maxwell’s equations and calculate the optical mode distributions and effective refractive index for both quasi-TE and quasi-TM polarizations at both pump operative wavelengths. It is worth outlining that the procedure is based on a multiphysics approach, i.e. the FEM electromagnetic module used in this step works together with the FEM mechanical module in order to perform the overlap integrals given in Eqs (5–7). Moreover, the FEM mechanical solver looks for solutions to the weak form of the partial differential equation (PDE) Eq. (3), which is an integral form of the original PDE. It is obtained by multiplying the original PDE with a test function and then integrating over the entire structural domain. Generally, the FEM mechanical module yields numerous solutions to Eq. (3), many of which do not contribute significantly to the acousto-optic scattering process. Thus, the selected Brillouin-active acoustic modes are those responsible for various significant peaks in the overall acoustic-optic overlap *Q*. However, for many applications, it is opportune to focus attention on the Brillouin-active acoustic mode responsible for the maximum peak. Finally, the home-made code, is used for data processing.

Our approach for calculating the acoustic modes of the waveguide is as follows: The mechanical eigenvalue problem has been implemented using the COMSOL package in 2-D space in the general Partial Differential Equations (PDE) Modes, wherein the solver looks for solutions to the weak form of the PDE, which is an integral form of the original PDE. The standard boundary conditions are then applied: continuity across all the interfaces of the three components of the displacement vector, normal compressional stress, and shear stress. In addition, the natural or stress-free conditions have been assumed on the outer interfaces, meaning that the total normal stress vanishes on the outer boundary of the structures^40,41^.
